# Multiomic analyses reveal new targets of polycomb repressor complex 2 in Schwann lineage cells and malignant peripheral nerve sheath tumors

**DOI:** 10.1093/noajnl/vdae188

**Published:** 2024-11-09

**Authors:** Minu M Bhunia, Christopher M Stehn, Tyler A Jubenville, Ethan L Novacek, Alex T Larsson, Mahathi Madala, Suganth Suppiah, Germán L Velez-Reyes, Kyle B Williams, Mark Sokolowski, Rory L Williams, Samuel J Finnerty, Nuri A Temiz, Ariel Caride, Aditya V Bhagwate, Nagaswaroop K Nagaraj, Jeong-Heon Lee, Tamas Ordog, Gelareh Zadeh, David A Largaespada

**Affiliations:** Department of Genetics, Cell Biology and Development, University of Minnesota, Twin Cities, Minneapolis, Minnesota, USA; Department of Pediatrics, Masonic Cancer Center, University of Minnesota, Minneapolis, Minnesota, USA; Department of Genetics, Cell Biology and Development, University of Minnesota, Twin Cities, Minneapolis, Minnesota, USA; Department of Pediatrics, Masonic Cancer Center, University of Minnesota, Minneapolis, Minnesota, USA; Department of Pediatrics, Masonic Cancer Center, University of Minnesota, Minneapolis, Minnesota, USA; Department of Pediatrics, Masonic Cancer Center, University of Minnesota, Minneapolis, Minnesota, USA; Department of Pediatrics, Masonic Cancer Center, University of Minnesota, Minneapolis, Minnesota, USA; Department of Pediatrics, Masonic Cancer Center, University of Minnesota, Minneapolis, Minnesota, USA; MacFeeters-Hamilton Center for Neuro-Oncology, Princess Margaret Cancer Center, Toronto, Ontario, Canada; Division of Neurosurgery, University Health Network, University of Toronto, Toronto, Ontario, Canada; Department of Pediatrics, Masonic Cancer Center, University of Minnesota, Minneapolis, Minnesota, USA; Department of Pediatrics, Masonic Cancer Center, University of Minnesota, Minneapolis, Minnesota, USA; Department of Pediatrics, Masonic Cancer Center, University of Minnesota, Minneapolis, Minnesota, USA; Department of Pediatrics, Masonic Cancer Center, University of Minnesota, Minneapolis, Minnesota, USA; Department of Genetics, Cell Biology and Development, University of Minnesota, Twin Cities, Minneapolis, Minnesota, USA; Department of Pediatrics, Masonic Cancer Center, University of Minnesota, Minneapolis, Minnesota, USA; Department of Pediatrics, Masonic Cancer Center, University of Minnesota, Minneapolis, Minnesota, USA; Epigenomics Development Laboratory, Epigenomics Program, Center for Individualized Medicine, Mayo Clinic, Rochester, Minnesota, USA; Department of Biomedical Statistics and Informatics, Mayo Clinic, Rochester, Minnesota, USA; Department of Biomedical Statistics and Informatics, Mayo Clinic, Rochester, Minnesota, USA; Epigenomics Development Laboratory, Epigenomics Program, Center for Individualized Medicine, Mayo Clinic, Rochester, Minnesota, USA; Epigenomics Development Laboratory, Epigenomics Program, Center for Individualized Medicine, Mayo Clinic, Rochester, Minnesota, USA; MacFeeters-Hamilton Center for Neuro-Oncology, Princess Margaret Cancer Center, Toronto, Ontario, Canada; Division of Neurosurgery, University Health Network, University of Toronto, Toronto, Ontario, Canada; Department of Genetics, Cell Biology and Development, University of Minnesota, Twin Cities, Minneapolis, Minnesota, USA; Department of Pediatrics, Masonic Cancer Center, University of Minnesota, Minneapolis, Minnesota, USA

**Keywords:** MPNST, NF1, nirogacestat, notch signaling, PRC2

## Abstract

**Background:**

Malignant peripheral nerve sheath tumors (MPNSTs) can arise from atypical neurofibromas (ANF). Loss of the polycomb repressor complex 2 (PRC2) is a common event. Previous studies on PRC2-regulated genes in MPNST used genetic add-back experiments in highly aneuploid MPNST cell lines which may miss PRC2-regulated genes in *NF1*-mutant ANF-like precursor cells. A set of PRC2-regulated genes in human Schwann cells (SCs) has not been defined. We hypothesized that PRC2 loss has direct and indirect effects on gene expression resulting in MPNST, so we sought to identify PRC2-regulated genes in immortalized human Schwann cells (iHSCs).

**Methods:**

We engineered *NF1*-deficient iHSCs with loss of function *SUZ12* or *EED* mutations. RNA sequencing revealed 1327 differentially expressed genes to define PRC2-regulated genes. To investigate MPNST pathogenesis, we compared genes in iHSCs to consistent gene expression differences between ANF and MPNSTs. Chromatin immunoprecipitation sequencing was used to further define targets. Methylome and proteomic analyses were performed to further identify enriched pathways.

**Results:**

We identified potential PRC2-regulated drivers of MPNST progression. Pathway analysis indicates many upregulated cancer-related pathways. We found transcriptional evidence for activated Notch and Sonic Hedgehog (SHH) signaling in PRC2-deficient iHSCs. Functional studies confirm that Notch signaling is active in MPNST cell lines, patient-derived xenografts, and transient cell models of PRC2 deficiency. A combination of MEK and γ-secretase inhibition shows synergy in MPNST cell lines.

**Conclusions:**

We identified PRC2-regulated genes and potential drivers of MPNSTs. Our findings support the Notch pathway as a druggable target in MPNSTs. Our identification of PRC2-regulated genes and pathways could result in more novel therapeutic approaches.

## Lay Summary

Malignant peripheral nerve sheath tumors (MPNSTs) are cancers that affect the protective covering of nerves. In these cancers, a protein complex called PRC2, which is important for normal cell behavior, is often absent. The authors of this study wanted to figure out how the loss of the PRC2 may cause tumors to grow. To do this, they studied normal cells that cover nerves that lack PRC2 and compared them with MPNSTs. Their results showed that losing PRC2 changes many genes, including some that are linked to cancer development. Two important pathways, called Notch and SHH, were found to play a key role. Blocking these pathways helped slow down tumor growth.

**Key Points**

- PRC2 loss in iHSCs leads to 1327 differentially expressed genes and altered during MPNST genesis.
- SHH and Notch signaling pathways are activated in engineered PRC2-deficient iHSCs.
- Combination γ-secretase and MEK inhibition may be effective for MPNSTs.

**Importance of the Study**

We describe a PRC2-regulated gene set in engineered human *NF1*-deficient SCs, which are relevant to ANF progression to MPNST. These CRISPR-generated mutant cell lines have not been previously studied for gene expression, DNA methylation, proteomic, and histone modification changes. We confirmed previous reports of RAS-MAPK and SHH-regulated gene upregulation in *NF1* and PRC2-deficient SCs. We identified other PRC2-regulated pathways, including Notch signaling, which have not been thoroughly investigated in MPNST genesis. The recently FDA-approved γ-secretase inhibitor nirogacestat, which blocks Notch activation and is useful to treat desmoid tumors, inspired further studies of this drug as a potential treatment for MPNST. We found nirogacestat is synergistic in MPNSTs with the MEK inhibitor, mirdametinib. We demonstrate that Notch signaling is an important target for further study in NF1 and MPNST research.

Neurofibromatosis Type 1 (NF1) is an autosomal dominant cancer predisposition syndrome. Roughly half of NF1 patients develop plexiform neurofibromas (PN), benign tumors partially containing neoplastic *NF1*-deficient Schwann cells (SC). Benign PNs can progress to atypical neurofibromas (ANF), and then to malignant peripheral nerve sheath tumors (MPNSTs).^1^ PNs develop after somatic loss of the wild-type *NF1* allele resulting in an increase in Ras-GTP activated signaling as NF1 encodes the important Ras GTPase activating protein neurofibromin.^2^ The ANF stage often exhibits biallelic loss of *CDKN2A/B* expression.^3^ MPNSTs have been characterized by profound chromosome instability, gain of chromosome 8, activation of *TERT* or alternative lengthening of telomeres, and other gene copy number changes.^4^ Loss of the polycomb repressor complex 2 (PRC2) occurs in more than 80% of MPNSTs via biallelic inactivation of either *SUZ12* or *EED*.^1^ This results in the loss of histone H3 lysine 27 trimethylation (H3K27me3), a repressive mark that silences genes through the formation of heterochromatin.^5^ Several mechanisms have been proposed for how PRC2 loss drives oncogenesis in MPNST. PRC2 inactivation has been found to derepress Ras-MAPK–dependent gene expression in a glioma cell line, whereas *SUZ12* restoration repressed Ras-MAPK–dependent gene expression in an MPNST cell line.^6^ Recent findings indicate that PRC2 loss causes reduced expression of HLA class I, antigen presentation, and type I interferon pathway genes.^7,8^ Using SUZ12 reexpression strategies, 2 groups have shown PRC2 loss likely derepresses expression of a suite of developmentally regulated transcription factors like *HOXB8* and *MSX1/2* inducing a mesenchymal neural crest-like phenotype.^1,9^ Sonic hedgehog (SHH) pathway activation has been associated with PRC2-deficient MPNSTs and defines a proposed molecular subgroup.^10^ Though implicated in SC development and targeted for desmoid tumor treatment, Notch signaling has not been well studied in MPNSTs.^11,12^ In normal brain glial cell development, the PRC2 has been shown to repress Notch signaling to regulate the balance between oligodendrocyte and astrocyte production.^13^ We hypothesized that a careful discovery of PRC2-regulated genes in immortalized human Schwann cells (iHSCs) would uncover more PRC2 derepressed genes and pathways providing new druggable targets. We performed RNA sequencing (RNA-seq), methylome, proteomic, and chromatin immunoprecipitation sequencing (ChIP-seq) analyses to investigate iHSCs with loss of functional NF1 and PRC2 and compared these results with gene expression differences between ANF and MPNST patient samples. Pathway analysis revealed that SHH and Notch signaling pathways are activated in engineered PRC2-deficient iHSCs.

To determine which PRC2-regulated changes in gene expression might be operative in human MPNST, we compared gene expression differences between human ANF, which are PRC2 intact, and MPNSTs. To further refine direct and indirect targets of PRC2, these gene expression changes were compared with genes that are altered when *SUZ12* is reexpressed in MPNST cell lines and genes with strong promoter enrichment of H3K27me3 in PRC2-intact iHSC.^9^ We validated our findings via Western blotting, qPCR, and small molecule studies on various cell line models.

## Materials and Methods

### Ethics Statement

This study did not involve human subjects but utilized genomics data derived from de-identified patient samples. All animal studies were conducted in accordance with the guidelines set forth by the University of Minnesota and federal regulations. The protocols for animal use were approved by the Institutional Animal Care and Use Committee (IACUC) of the University of Minnesota.

### Cell Lines and Reagents

We received iHSCs from Dr Margaret Wallace (University of Florida, Gainesville). These are wild-type human neonatal SCs immortalized using the human reverse transcriptase component of telomerase (hTERT) transgenesis and overexpression of murine cyclin–dependent kinase (Cdk4).^14^ To improve modeling ANF and MPNST stages, we stably knocked out *NF1* (discussed in Williams et al.), *SUZ12*, and *EED* using CRISPR/Cas9 gene editing methods (Williams et al., unpublished manuscript).^15^ We used the published cotransposition method using *NF1* and *SUZ12* guide RNAs or transient Cas9/gRNA ribonucleoprotein complexes for *EED.*^16^ Single-cell clones were validated using Western blotting and Synthego’s Inference of CRISPR Edits analysis tool (https://ice.synthego.com). We also utilized immortalized PN hTERT NF1 ipNF95.6 (iPNF) and MPNST cell lines (sNF96.2 and sNF02.2) from American Type Culture Collection (ATCC) with loss of function *NF1* mutations. Other MPNST cell lines and patient-derived xenografts (PDX) were kindly provided by Dr Nancy Ratner (Cincinnati Children’s Hospital) (26T, S462TY, ST88-14, MPNST724), the Characterized Cell Line Core Facility (MD Anderson) (MPNST007), and Dr Christine Pratilas (Johns Hopkins) (JH-2-055 CL, JH-2-079c CL, JH-2-003 CL, JH-2-002 CL).^17–19^ Cell lines and media are listed in Supplementary Table S1.

### Sample Preparation and RNA-seq

Cell pellets of 3-4 million cells were collected. RNA was extracted using the RNeasy Mini Kit (Qiagen) and sent to the University of Minnesota Genomics Center. Sequencing was performed on a NovaSeq 6000 (Illumina) generating approximately 20 million reads/sample with 150 bp paired end reads.

### Differential Gene Expression Analysis and Pathway Analysis

RNA-seq data (https://www.ncbi.nlm.nih.gov/geo/query/acc.cgi?acc=GSE263107) was trimmed and aligned with Trimmomatic and HISAT2 to the human genome (hg38). Reads were counted using featureCounts.^20^ Differentially expressed genes were identified using DESeq2.^21^ Inclusion criteria for differentially expressed genes were an absolute fold change of normalized read counts in pairwise comparison greater than 2 with a false discovery rate (FDR) less than 0.05. Pathway analysis was performed using EnrichR and gene set enrichment analysis (GSEA) as previously described with the R package, *clusterProfiler*.^22–24^ Significantly enriched pathways scored a *P*-value less than .05 and FDR less than 0.25. Transcriptome files for patient samples were provided by our collaborator.^10^

### Illumina Infinium MethylationEPIC BeadChip Array and Analysis

Genome-wide DNA methylation was investigated using the Infinium methylome EPIC array platform (Illumina) at the University of Minnesota Genomics Center. Quality control, filtering, normalization, and statistical testing were performed as previously described.^25^ Differential methylation of probes was identified with *limma*. Filtering and normalization were performed using *minfi*. Average methylation of each gene was constructed using an average of beta values of each probe in the promoter region. Figure 2 uses the average difference between gene statuses (experimental—control).

### Sample Preparation and Global Proteomic Analysis by Liquid Chromatography-Mass Spectrometry

Multiplexed deep-scale proteome analysis by liquid chromatography-mass spectrometry (LC-MS/MS) was performed at the Broad Institute (Cambridge, MA) as previously described.^26^ Two replicates of 8 lines were used (Supplementary Table S1). Protein was extracted using an 8 M urea solution. Reduction, alkylation, lysis, and digestion steps were performed before tandem mass tag isobaric labeling of peptides. Labeled peptides were run through basic reversed-phase chromatography columns. Five percent of each fraction was collected for proteome analysis by LC-MS/MS. Data were analyzed with Spectrum Mill MS Proteomics Workbench. Differential protein abundance analysis was performed using the R package, *limma*.^27^

### Sample Preparation and ChIP-seq Analysis

Four million cells per histone mark were crosslinked with 1% formaldehyde (10 min) and quenched with 125 mM glycine (5 min, room temperature). Fixed cells were shipped to the Epigenomics Development Laboratory (Mayo Clinic). Lysed cells were treated with 100 gel units of MNase in MNase digestion buffer (20 min, 37 °C, continuous thermal mixing). Following sonication in Diagenode Bioruptor pico, the supernatant was subjected to ChIP with antibody overnight. ChIP and library preparation followed previous protocols.^28^ Libraries were sequenced on an Illumina NovaSeq 6000 platform at the Mayo Clinic Center for Individualized Medicine Medical Genomics Facility. Antibodies, primers, and buffers are detailed in Supplementary Table S1. Analysis was conducted in-house and at the Mayo Clinic Bioinformatics Core.^29^

### Western Blot Analysis

Cell lines were probed for various Notch pathway components, SUZ12, and controls with antibodies from Cell Signaling Technologies (Supplementary Table S1). Immunoblotting was conducted as previously described.^30^ Blots were imaged on an Odyssey Fc using Image Studio software (LI-COR). CUTLL1 was used as positive controls for NOTCH1, MDA-MB-231 for NOTCH1, NOTCH2, and NOTCH4, and MCF7 for HES1 and NOTCH3.

### Generation and Use of Transient iHSCs

#### Short interfering RNA treatment—

All treatments used SMARTpool ON-TARGETplus Human short interfering RNA (siRNA) (Dharmacon). Transient models of SUZ12 loss were created in iPNF and N0(32) cells using SUZ12 siRNA (5 nmol). Transient models of Notch pathway inhibition were created in ST88-14 cells using NOTCH1, 2, 3, and 4 siRNA (10 nmol). Nontargeting Control siRNA (20 nmol) was used as the control. We followed the manufacturer’s protocol (Dharmacon, 23512) in a 6-well format.

#### EZH2 inhibitor—

To simulate transient PRC2 loss, 350000 iPNF and N0(32) cells were treated with 1 µM EZH2 methyltransferase inhibitor (Selleck Chemicals, GSK126) for 10 days before harvesting cell pellets for Western blot.

### siRNA Proliferation Assay

On day 0, 350000 ST88-14 cells were plated in 6-well plates in triplicate. On day 1, cells received siRNA (NOTCH1, 2, 3, 4) treatments in serum-free media. Complete media was added to 2 wells on day 2. One well was seeded into a 96-well plate (5000 cells/well). The 96-well plate was stained with alamarBlue (Invitrogen) to determine proliferation on day 3. Fluorescence measurements were normalized to nontreated control (siNT).

### Gain of Function Cell Line Generation

Gain of function (GOF) lines of *NOTCH1* and *NOTCH3* were made in ST88-14, S462TY, iPNF, and N0(32) target cells using lentiviral transduction. Plasmids are listed in Supplementary Table S1.^31^ Lentiviral particles were generated as previously described.^32^ Cells were selected with puromycin.

### Transwell Migration Assay

Performed as previously described.^10^ Cells in serum-free media were seeded into wells containing serum media and 8 µm size pore transwell inserts. After 24 hours, inserts were stained with 0.1% crystal violet, imaged with a brightfield microscope, and manually counted.

### 2D Proliferation Assay

In 6-well plates, 20000 GOF cells were plated in duplicate. Cell counts were taken daily with a Countess for 5 days and plotted using GraphPad Prism software. Two-way ANOVA was used to determine significant differences in cell count between gene statuses.

### Quantitative RT-PCR

RNA was extracted using the PureLink RNA Mini Kit (Invitrogen). cDNA was synthesized using the SuperScript IV First Strand Synthesis Kit (Invitrogen). Reverse transcription, qPCR reactions, and analysis were performed as previously described.^30^ The dye used was FastStart SYBR Green Master (Sigma-Aldrich). qPCR reaction was performed on a CFX96 Touch Real-Time PCR Detection System (Bio-Rad). Primer sequences are listed in Supplementary Table S1.

### Tumor Microarray Visual Grading

Tumor microarray (TMA) blocks were created using patient-derived NF1 tissue samples (IRB #5384) and graded via hematoxylin and eosin (H&E) staining. Duplicate 1.0-mm cores were manually arrayed (max 64 cores/block) and processed by HistoWiz. Deparaffinized 4 μm TMA sections underwent standard rehydration and heat antigen retrieval. Staining involved the peroxidase method, hematoxylin counterstaining, dehydration, citrosolv clearing, and Permount mounting. Sections were assessed for tumor sampling, confirming duplicates contained tumor tissue on H&E staining. No-stain and internal negative controls ensured no staining intensity. DAB staining intensity was visually scored (0 = no stain, 1 = low, 2 = medium, 3 = high) independently by G.L.V. following visual training, as described.^33^

### Small Molecule Inhibitor Study

MPNST cell lines (wild-type and GOF) were treated with γ-secretase inhibitor, nirogacestat (Selleckchem, S8018), and mirdametinib (Selleckchem, S1036) in 384-well format for 48 h at the Institute for Drug Discovery and Therapeutics (University of Minnesota). Plating density was 2000 cells/well using a Biomek 2000 (Beckman Coulter). The following day cells received the drug in 12, 2-fold dose format using a LabCyte Echo 550 (Beckman Coulter). Two days later, viability was quantified using alamarBlue according to the manufacturer’s protocol (Invitrogen) and fluorescence read on a CLARIOstar microplate reader (BMG Labtech). Viability was calculated as previously described (Williams et al., unpublished manuscript).^15^ Drug combinations were in constant ratios. Dose-response curves were generated and IC_50_ was determined using Graphpad Prism software using a nonlinear regression log(inhibitor) vs response-variable slope model. Combination index values were determined with the median-effect principle using CalcuSyn software (Biosoft).^34^

### Flow Cytometry

ST88-14 were treated with nirogacestat for 48 h. Following treatment, cells were stained with the Dead Cell Apoptosis Kit with Annexin V Alexa Fluor™ 488 & Propidium Iodide following the manufacturer’s protocol (Invitrogen). Cells were also stained with Vybrant™ DyeCycle™ Violet Stain following the manufacturer’s protocol (Invitrogen). Data were acquired using a CytoFLEX flow cytometer (Beckman Coulter) and 10 000 single cells were analyzed per sample. Data were analyzed using FlowJo.

## Results

### Global Gene Expression Changes Occur After PRC2 Loss in iHSCs

PRC2-regulated genes that may drive MPNSTs have primarily been defined by comparing PRC2-deficient versus proficient MPNSTs or by reexpression of *SUZ12* in 1 or 2 PRC2-deficient MPNST cell lines.^1,6,9^ To define a set of PRC2-regulated genes in human Schwann lineage cells and discover PRC2-repressed genes that may drive MPNST genesis, we engineered novel CRISPR/Cas9-mediated knockout human SCs. These lines harbor mutations in *NF1* and 1 component of PRC2 (*SUZ12* or *EED*). To investigate the differential expression of iHSCs, *NF1*-deficient only samples were used as controls to assess the contribution of loss of PRC2 on a background of *NF1* deficiency (Supplementary Figure S1). For patient samples, ANF was the control. Various comparisons were used to investigate genes derepressed by loss of PRC2 in iHSCs and dysregulated during the transition from ANF to MPNSTs (Supplementary Table S1).

**Figure 1.**
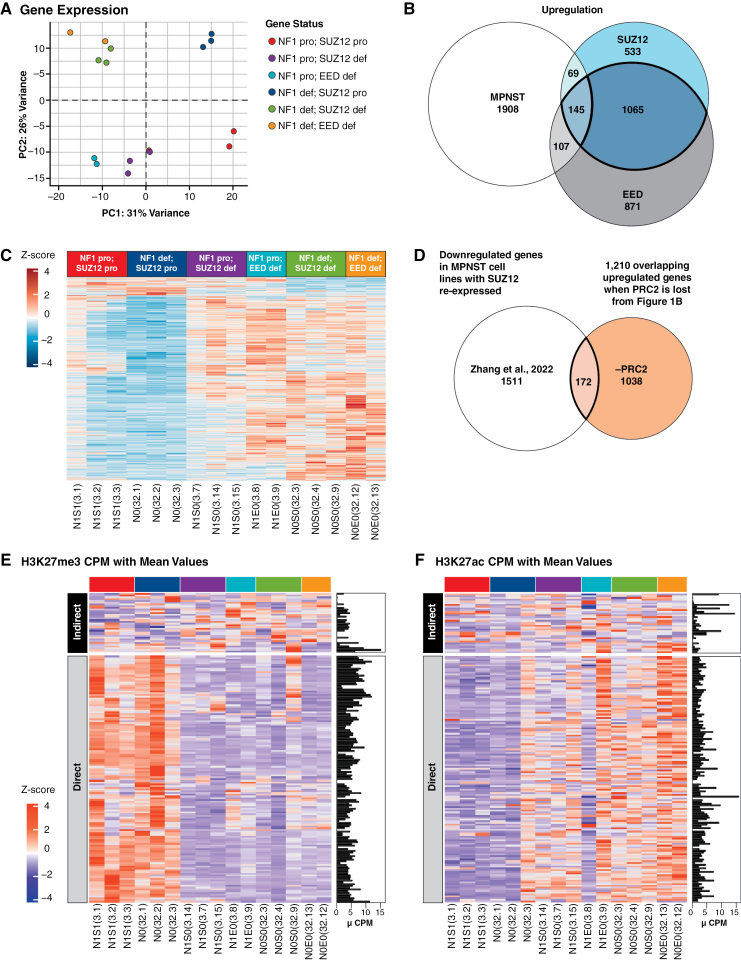
Differential expression analyses reveal 1327 genes that are altered by PRC2 status in *NF1*-deficient human Schwann cell (SCs), are upregulated or downregulated during transformation, and are potential mediators of the MPNST phenotype. PCAs of engineered immortalized human Schwann cell (iHSC) lines using (A) gene expression data. (B) Overlap of differentially upregulated genes from MPNSTs, *NF1* and PRC2 (*SUZ12* or *EED*)-deficient human SCs leads to 145 upregulated differentially expressed genes; 1210 genes are upregulated when PRC2 is lost (shown in bolded overlap). (C) Heatmap of overlapping 1327 differentially expressed, PRC2-regulated genes across all engineered human SCs. Values are gene-wise *Z* scores. (D) Overlap of downregulated differentially expressed genes from MPNSTs with PRC2 restored from Zhang et al. and 1210 upregulated genes from iHSCs. Heatmaps of the (E) H3K27me3 and (F) H3K27ac status of 172 direct and indirect targets of PRC2. The downregulation Venn diagram is shown in Supplementary Figure S2C. All Western blot quantification can be found in Supplementary Figure S6.

We performed RNA-seq on engineered iHSCs and generated a principal component analysis (PCA) plot using the top 500 variant genes and found clear clusters by *NF1* and PRC2 statuses (Figure 1A).

### PRC2 Represses Thousands of Genes in Human SCs

Loss of *NF1* alone resulted in 1439 differentially expressed genes compared with wild-type cells (Supplementary Figure S2A). *EED* knock out on an *NF1*-deficient background resulted in 2521 differentially expressed genes compared with *NF1*-deficient alone. *SUZ12* knock out on an *NF1*-deficient background resulted in 2234 differentially expressed genes compared with *NF1* knockout alone. Loss of PRC2 function caused a greater increase in the number of differentially expressed genes than loss of *NF1* alone. Most genes whose expression changed after the loss of *SUZ12* or *EED* on an *NF1*-deficient background, 81% and 87%, respectively, were upregulated compared with *NF1*-deficient cells alone, consistent with the known role of PRC2 in transcriptional repression (Figure 1B and C and Supplementary Figure S2B, Table S2).

### Promoter Methylation Status of Genes Upregulated in iHSCs After PRC2 Inactivation Is Not Usually Altered and Many Upregulated Genes Show Promoter Hypermethylation

**Figure 2.**
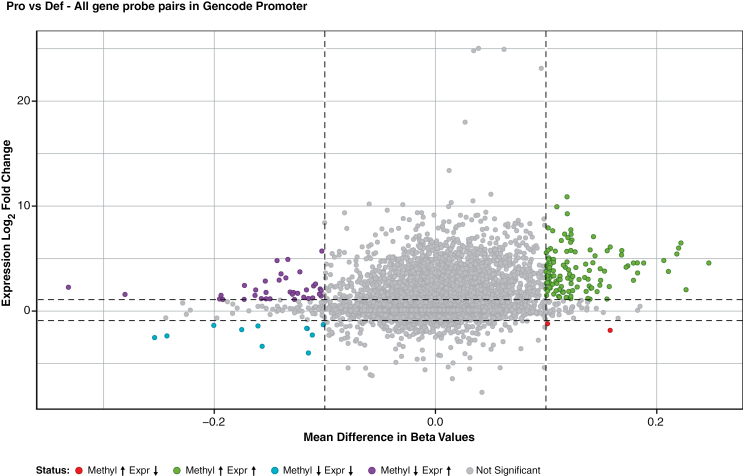
Genes derepressed by PRC2 inactivation do not show consistent changes in promoter methylation. Integrative analysis of DNA methylation and gene expression of engineered cell lines using a scatterplot of mean difference of beta values versus log2 expression fold change. Each point represents a gene-probe pair of the engineered lines. Adapted from Xu et al.^35^

We compared PRC2-deficient iHSC methylation and expression data. Methylation data were plotted as mean difference of beta values, whereas gene expression was shown as a log2fold change of expression. The significance cutoffs were 0.1 magnitude mean difference in beta values and log2fold change of 1. All gene probe pairs in promoter regions of iHSCs were compared (Figure 2).^35^ We found that most genes upregulated by genetic inactivation of PRC2 do not show concomitant changes in promoter methylation, neither becoming more hypomethylated nor hypermethylated.

Others have reported PRC2 influences genome methylation patterns so we performed the Infinium 850K MethylationEPIC array (Illumina) on iHSCs.^8^ We plotted a PCA using methylation data (Supplementary Figure S2C). We did not observe notable methylation changes between gene states. We saw no consistent changes in repeat element methylation (Alu, LINE1, endogenous retroviruses, and all repeats combined) with loss of PRC2 (data not shown).

### Proteomic Analysis Reveals NF1 and PRC2-Regulated Proteins

To discover proteins whose level of expression changed after genetic inactivation of *NF1* and PRC2, we performed LC-MS/MS proteomic analyses on a subset of iHSCs. With moderate correlation between replicates, we focused on changes in protein levels of the products of the 1327 genes that had consistent changes in mRNA levels in *EED* and *SUZ12* knockouts. This analysis showed 354 genes were present in the proteome data (Supplementary Figure S2D, Table S2). We observed an increased abundance of Notch (PSEN2) and Ras (KSR1) signaling, as well as other proteins, in PRC2-deficient clones. BCL11B prevents senescence and apoptosis due to hyperactive Ras signaling.^36^ AK4 activates HIF1A and promotes epithelial-mesenchymal transition (EMT) under hypoxia and can reduce viability when knocked out in cancer cells.^37^ CD70 increases regulatory T-cell survival in tumors and is a potentially useful immunotherapy target.^38^ In all knockout lines, the most differentially abundant protein was the target of the knockout (data not shown).

### Genetic Inactivation of PRC2 in Human SCs Derepresses Known PRC2 Targets

Very few curated (C2) gene sets specifically regarding PRC2 target genes are listed in the Molecular Signatures Database (https://www.gsea-msigdb.org/gsea/msigdb/human/genesets.jsp?collection=C2). But we observed published gene sets, either directly related to PRC2 targets or indirectly regarding H3K27me3, to be upregulated in *NF1* and PRC2-deficient iHSCs, including PASINI_SUZ12_TARGETS_UP from a study on mouse embryonic stem cells with loss of *Suz12*^39^ and a study of prostate cancer cells after *EZH2* knockdown (KONDO_EZH2_TARGETS).^40^

Among upregulated genes were several homeobox cluster genes (*HOXB8* and *MSX1/2*), consistent with extensive literature describing PRC2 regulation of *HOX* genes in development.^9,41^ GSEA of upregulated genes in PRC2-deficient iHSCs revealed strong overlap with a set of genes characterized by high CpG content in their promoters that are also enriched for H3K27me3 in mouse neural progenitor cells (MIKKELSEN_NPC_HCP_WITH_H3K27ME3), suggesting they are PRC2 targets in this cell type.^42^ These findings suggest our iHSCs reveal true PRC2-regulated pathways (Supplementary Table S3).

### PRC2 Loss Amplifies RAS-MAPK-Dependent Gene Expression

Increases in RAS-MAPK-dependent gene expression after PRC2 loss have been described and hypothesized to contribute to malignant transformation to MPNST.^2,6^ GSEA, using Hallmark gene sets, revealed genes upregulated by KRAS activation (HALLMARK_KRAS_SIGNALING_UP) and downregulated by KRAS activation (HALLMARK_KRAS_SIGNALING_DN) enriched in *NF1* and PRC2-deficient lines compared with *NF1*-deficient only lines (Supplementary Table S3). These data are consistent with the hypothesis that loss of PRC2 function amplifies gene expression changes downstream of RAS-MAPK signaling.

### Genetic Inactivation of PRC2 in NF1-Deficient iHSCs Derepressed Human Immune Signature Genes

Other groups have reported IFN-γ signaling, antigen presentation, and regulation of MHC class I and II expression which are markedly reduced when PRC2 is lost.^7^ Some suggest PRC2 regulates these genes and decreases tumor immunogenicity.^8^ GSEA with Gene Ontology (GO) terms revealed these previously identified were found to be upregulated in our PRC2-deficient engineered iHSCs, such as adaptive immune response, B-cell receptor signaling pathway, antigen binding, and activation of innate immune response (Supplementary Table S3).

### Genes Derepressed by Loss of PRC2 in Human SCs Include Direct and Indirect Targets and Potential Drivers of MPNST

To discover genes deregulated by loss of PRC2 function and relevant in MPNST genesis, we investigated PRC2 derepressed genes, in *NF1* and PRC2-deficient iHSCs, that are also significantly upregulated during the transition from MPNST. We observed 1210 upregulated genes, common to loss of *SUZ12* and *EED*, on an *NF1*-deficient background. Overlapping these with genes upregulated in human MPNST in comparison to ANF led to 145 genes (Figure 1B and Supplementary Table S2). We hypothesize these 145 genes are potential candidate drivers of MPNST. Some of these genes are reported in other cancer-implicated signaling pathways such as Ras (*SPON1*), SHH (*SMO*), and Notch (*HEY2*).

Zhang et al. performed RNA-seq on 3 MPNST cell lines after *SUZ12* doxycycline-regulated vector complementation.^9^ They reported 1683 genes downregulated when *SUZ12* is reexpressed in *SUZ12*-deficient MPNST cells. We overlapped these genes with the 1210 genes that were upregulated when PRC2 is lost in human *NF1*-deficient SCs and found 172 genes in common (Figure 1D and Supplementary Table S2). EnrichR results of these 172 overlap genes showed upregulation of KRAS signaling and cytokine-cytokine receptor interaction (data not shown). We also observed ChIP-seq gene sets of PRC2 components and H3K27me3. This means that many of our 172 genes may also be bound by PRC2. To determine which of these genes are likely to be direct targets of PRC2, we performed ChIP-seq on engineered iHSCs for the histone methylation mark H3K27me3 and acetylation mark H3K27ac. We hypothesized direct gene targets of PRC2 would show strong promoter (+/- 5 kb of the transcriptional start site) enrichment of H3K27me3 before *SUZ12* or *EED* knockout and strong H3K27ac after knockout of either gene. One hundred and thirty-nine (139) gene promoters (81%) were strongly H3K27me3 positive before the loss of PRC2, and strongly H3K27ac positive after the loss of PRC2, so are likely direct targets of PRC2. The remaining genes are likely indirect targets of PRC2 and had low H3K27me3 reads (Figure 1E and F and Supplementary Table S2).

### PRC2 Regulates Notch Pathway Genes in NF1-Deficient iHSCs and MPNSTs

**Figure 3.**
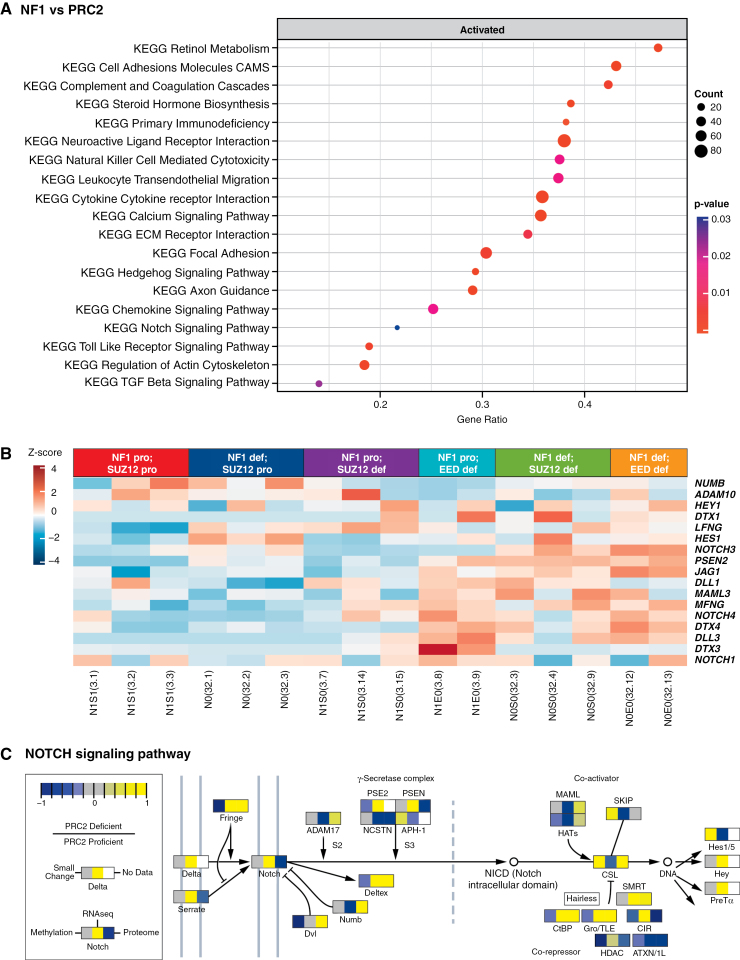
NOTCH signaling is a significantly upregulated pathway when PRC2 is lost in engineered iHSCs. Gene set enrichment analysis using clusterProfiler of iHSCs with (A) NF1 and PRC2 loss. (B) Gene expression heatmap of NOTCH signaling pathway players across all engineered lines. (C) NOTCH signaling pathway rendered by Pathview compiling RNA seq (middle panel), methylome (left panel), and proteome (right panel) data representing the ratio in change of each signaling pathway player after PRC2 loss in iHSCs.

Our analysis of genes upregulated after the loss of the PRC2 in *NF1*-deficient iHSCs revealed the enrichment of several cancer-related pathways (Figure 3A and Supplementary Table S3). These include KEGG Cell Adhesion Molecules (*CADM3*, *NCAM1*, and *NECTIN1*), Extracellular Matrix (ECM) Receptor Interaction (*ITGA8* and *COL6A6*), Hedgehog Signaling Pathway (*SHH* and *SMO*), and Notch Signaling Pathways (*NOTCH3*, *NOTCH4*, *DLL1*, *DLL3*, *JAG1*, *MFNG*, *DTX3*, *DTX4*, and *PSEN2*) (Figure 3B). We combined RNA-seq, methylome, and proteomics into 1 Pathview to generate an integrated view of Notch signaling (Figure 3C).^43^

**Figure 4.**
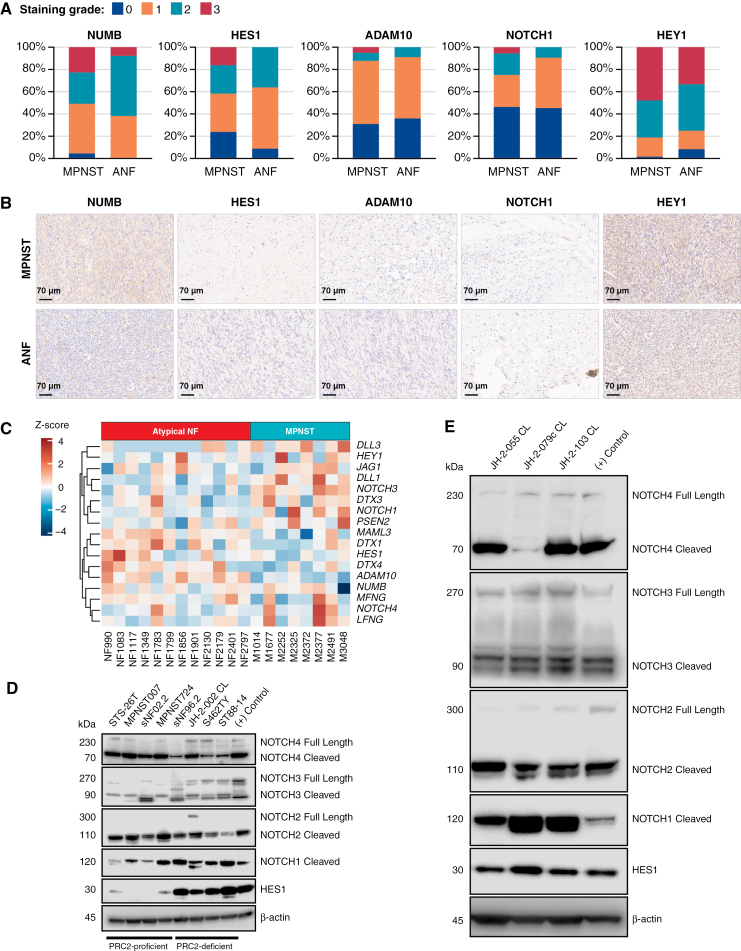
Gene expression analysis of patient ANF and MPNSTs reveals activation of the Notch pathway. (A) Visual grading of various NOTCH pathway effectors from TMA data using immunohistochemistry. (B) Representative images of TMA staining. The scale bar is 70 µm. All shown MPNSTs are scored as intensity 3 and ANFs are 0-1. Other examples of staining intensity can be found in Supplementary Figure S5. (C) Gene expression heatmap of NOTCH signaling pathway players across ANF (*n* = 12) and MPNST (*n* = 8) patient samples. Notch pathway players were probed in multiple (D) MPNST and (E) PDX cell lines. (+) control refers to MDA-MB-231 cells for all marks except HES1 and NOTCH3 where MCF7 cells were used.

Next, we analyzed primary ANF and MPNST using immunohistochemistry of a TMA to observe changes in Notch signaling (Figure 4A and B). Immunohistochemistry of NUMB, HES1, ADAM10, NOTCH1, and HEY1 revealed an increase in Notch pathway activation in some MPNSTs compared with premalignant precursors. We acquired patient ANF and MPNST data from a study reporting MPNSTs cluster into 2 distinct groups, SHH-like and WNT-like.^10^ Differential expression analysis of a subset of 20 samples comparing ANFs to MPNSTs found 2489 differentially expressed genes (data not shown). Notch pathway expression across ANF and MPNST samples had no clear, consistent pattern after malignant transformation, but certain MPNSTs showed consistent upregulation of NOTCH players (Figure 4C).

Because we observed changes in Notch signaling transcript abundance after PRC2 inactivation in SCs, we investigated Notch signaling in MPNSTs and transient knockdown cell models. Using Western blotting of cleaved (active) and full length (inactive) versions of all 4 NOTCH receptors, we found cleaved NOTCH in multiple MPNSTs and higher HES1 expression in PRC2-deficient MPNSTs compared with PRC2-intact MPNSTs (Figure 4D). Following this logic, Notch signaling was also active in multiple PRC2-deficient, early passage PDX-derived lines (Figure 4E).

**Figure 5.**
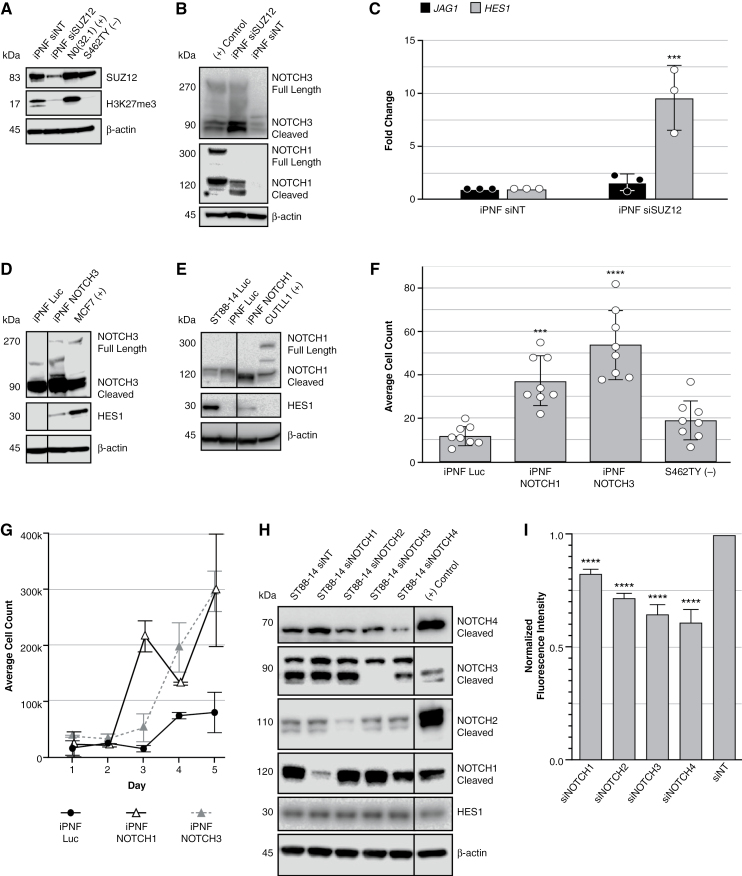
NOTCH signaling modulates growth behaviors in transient PRC2 knockdown Schwann cell models and PRC2-deficient MPNSTs. (A) Western blots to confirm SUZ12 was knocked down in iPNF cells. (B) NOTCH1 and NOTCH3 were probed in a transient SUZ12 knockdown in iPNF cells. (+) control refers to CUTLL1 cells for NOTCH1 and MCF7 cells for NOTCH3. (C) RT-qPCR of upstream and downstream markers of NOTCH signaling on generated transient cell line models (pooled siRNA). (D, E) Western blots to confirm successful transfection of GOF vectors of Notch pathway players (*NOTCH1*, *NOTCH3*, and Luciferase control) in iPNF lines. (F) Average cell counts of GOF cell lines following 24-h transwell migration assay. (G) Proliferation of iPNF GOF lines in a 6-well plate was tracked over 5 days; 20 000 cells were plated on day 0. Two-way ANOVA of Luc vs *NOTCH1* yields a *P*-value of .004 and Luc vs *NOTCH3* yields a *P*-value of .0143. (H) Western blots to confirm siRNA knockdown of *NOTCH* receptors in MPNST cell lines. (I) Proliferation of siRNA knockdown MPNST cell lines with AlamarBlue in 96-well plate format. ***P ≤ .001, ****P ≤ .0001.

To make transient models of PRC2 loss, we modulated *SUZ12* in SCs lacking *NF1* expression (iPNF) using RNA interference. iPNF cells are *TERT/Cdk4* immortalized, PN-derived SCs with loss of heterozygosity for *NF1*, express no neurofibromin protein, and thus model a precursor to MPNST. This naturally occurring line was chosen for downstream studies to mitigate effects from any other introduced edits. Transient knockdown of *SUZ12* (siRNA treatment every 2 days for 1 week) in iPNF significantly decreased SUZ12 protein and increased cleaved NOTCH1 and NOTCH3 via Western blot (Figure 5A and B). RT-qPCR analyses showed increased mRNA of the Notch ligand, *JAG1,* and downstream effector, *HES1*. *JAG1* mRNA was increased in EZH2 inhibitor–treated iPNF and N0(32) cells (Supplementary Figure S3A-C). *HES1* mRNA was significantly increased in siSUZ12-treated iPNF and N0(32) cells (Figure 5C and Supplementary Figure S3D and E). Suppression of PRC2 activity can activate NOTCH1 and NOTCH3 in immortalized *NF1*-deficient PNs, a process that might involve upregulation of Notch ligands like JAG1.

### Notch Signaling Regulates SC Proliferation and Migration

To determine if Notch signaling modulates MPNST cell growth, survival, and migration, we used siRNA and GOF vectors expressing the processed, cytoplasmic active intracellular domains (ICD) of NOTCH1 and NOTCH3. GOF cell lines for *NOTCH1* and *NOTCH3* were created in several *NF1*-deficient lines (Figure 5D and E and Supplementary Figure S3F-H). Ectopic expression of activated *NOTCH1* or *NOTCH3* drove 2D proliferation and cell migration in cells. Both GOF *NOTCH1* and *NOTCH3* transgene expression in iPNF, and *NOTCH3* expression in N0(32) cells, increased migration in a transwell assay (Figure 5F and Supplementary Figure S3I). To assess proliferation, cells were counted over 5 days. Day-to-day changes in iPNF and N0(32) proliferation were generally subtle, but GOF lines outgrew the control luciferase lines (Figure 5G and Supplementary Figure S3J). Transient siRNA treatment of Notch receptors (*NOTCH1-4*) in the PRC2-deficient MPNST line, ST88-14, was used to determine short-term changes in proliferation (Figure 5H). In a 48-h assay, the knockdown of *NOTCH* receptors individually decreased proliferation when compared with siNT (Figure 5I).

### Inhibitors of γ-Secretase and MEK Act Synergistically Versus MPNST

To determine if Notch signaling could be therapeutically targeted, alone and in combination with MEK inhibition, we performed preliminary small molecule inhibitor studies. We utilized the γ-secretase inhibitor, nirogacestat, recently FDA approved to treat desmoid tumors.^12^ We report average IC_50_ values of nirogacestat and the MEK inhibitor, mirdametinib, across various MPNST patient samples (Table 1 and Supplementary Figure S4A). At doses sufficient to inhibit MPNST growth, we observed inhibition of NOTCH1 and NOTCH3 processing to their active ICD cytoplasmic forms in ST88-14 cells at 70 µM (Supplementary Figure S4B). Nirogacestat and mirdametinib were synergistic in MPNST cell lines (Table 1). Next, to assess changes in cell death and apoptosis post 48-h nirogacestat treatment, we performed flow cytometry on ST88-14 cells. We observed an increase in early and late apoptosis in ST88-14 cells treated with the IC_50_ dose of nirogacestat. During cell cycle analysis, we identified a sub-G_1_ population compared with the control DMSO treatment (Supplementary Figure S4C). To determine if NOTCH processing inhibition is responsible for the inhibition of proliferation by nirogacestat, we determined IC_50_ in *NOTCH* GOF MPNST lines compared with controls. While GOF *NOTCH1* ICD expression could reduce the effectiveness of nirogacestat in ST88-14 cells, we did not observe this effect in S462TY cells (Supplementary Figure S4D).

**Table 1.** Combination Nirogacestat and Mirdametinib Treatments Are Synergistic in Immortalized Human Schwann Cells and MPNSTs

	IC_50_ (µM)		CI^a^			
Cell line	Mirda	Niro	ED50	ED75	ED90	*r*
26T	373.6	1215	0.23	0.18	0.14	0.90
MPNST007	178.5	1784	0.44	0.42	0.39	0.86
sNF02.2	312.2	760.3	0.090	0.036	0.015	0.67
MPNST724	340.9	999.9	0.29	0.25	0.21	0.94
sNF96.2	0.2343	28.89	0.083	0.12	0.19	0.84
JH-2-002 CL	347.5	112.3	0.34	0.46	0.66	0.95
S462TY	345.4	232.6	0.46	0.35	0.27	0.96
ST88-14	11.30	70.95	0.18	0.17	0.15	0.96

Abbreviations: IC_50_, average half maximal inhibitory concentration; Mirda, mirdametinib; Niro, nirogacestat; ED, effective dose; CI, combination index; *r*, fit value.

^a^Combinations are in constant ratio. Synergy scores were calculated using the CalcuSyn software and reported at 3 different effective doses. Generally, the cutoff for synergy are as follows: less than 1 indicates synergy, equal to 1 indicates additive, and greater than 1 indicates antagonistic.

## Discussion

We developed a novel iHSC model to explore *NF1* and PRC2 loss effects on biological pathways, valuable for mechanism, drug sensitivity, and synthetic lethality studies.^15^ Preferring a human cell model over the mouse, we aimed to capture subtle species-specific chromatin biology differences. With this loss of function system, we defined gene expression changes, revealing pathways potentially driving MPNST genesis after PRC2 loss. Notably, integrin-based cell adhesion pathways, SHH, and Notch signaling are regulated by PRC2 loss in human SCs.

We used a multiomic approach to characterize CRISPR engineered iHSCs and compared these to changes that accompany the transition from ANF to MPNST to focus on pathways likely to drive malignancy. Our analyses combined RNA-seq, methylome microarray, ChIP-seq, and mass spectrometry-based proteomics. Global gene expression analysis of our *NF1*-deficient iHSCs revealed 1327 differentially expressed genes after PRC2 inactivation, where 1210 genes are normally repressed by PRC2. A comparison with previously published genes that are repressed by reexpression of *SUZ12* in 2 MPNST cell lines, and genes enriched in H3K27me3 in human iHSCs, revealed 172 genes are potential direct targets of PRC2 and strong candidates for directly regulated, derepressed candidate drivers of MPNSTs in PRC2-deficient cases.^9^

We note the limitations of our study. The iHSCs in this study are immortalized cell lines generated from mature SCs. We generated a relatively small sample size (2-3 clones per gene status). The *EED* clones were also generated using a different method than the other iHSCs. To increase statistical power, we combined *SUZ12* and *EED*-deficient clones for a PRC2-deficient group for gene expression analysis. There is also the likelihood of genetic and epigenetic clonal drift to occur when cells are cultured leading to some differences between what should be isogenic clones.^44^ The MPNST patient samples are not all PRC2 deficient due to limited availability. Nevertheless, by providing a PRC2 loss of function model in human, *NF1*-deficient SCs, we have discovered new candidate driver pathways for MPNST.

We found that PRC2 represses regulators of the SHH pathway, like GLI1, in human NF1-deficient SCs. MPNSTs have been recently characterized into molecular subclasses. One subgroup is defined by SHH activation and promoter hypermethylation of *PTCH1*, whereas another group showed WNT pathway overexpression. These 2 clusters were characterized as being SHH-like (MPNST-G1) and WNT-like (MPNST-G2).^10^ Our findings indicate that the loss of PRC2 likely contributes directly to causing SHH activation in iHSCs, along with reduced expression of *PTCH1*.

We do not see evidence of global hypermethylation; however, enough individual CpG methylation sites change to distinguish genotypes seen in the methylome PCA. From this, we can speculate that PRC2 may regulate a subset of the methylome. We found upregulation of gene expression after the loss of PRC2 is not usually associated with consistent change in promoter DNA methylation status of most genes in human SCs. We suspect while loss of PRC2 does alter the DNA methylome, these alterations usually do not result in specific gene expression changes. Instead, changes to the epigenetic state of H3K27 are most responsible for changes in gene expression after PRC2 loss.^9^ We cannot rule out that such sequences are liable to DNA methylation inhibitor–induced changes in expression as others have shown in MPNSTs.^8^ Although proposed by others, we did not see consistent changes in methylation of endogenous retroviruses or transposable elements, after loss of PRC2 in *NF1*-deficient human SCs.^8^

In contrast to published findings, we see no evidence for repression of HLA class I gene expression or IFN-regulated pathways after loss of PRC2 in *NF1*-deficient iHSCs. Instead, many of these genes are derepressed by loss of PRC2. For example, chemokines *CXCL10* and *CCL5* were upregulated in PRC2-deficient clones with Log2fold change of 5.7 and 6.0, respectively. It is possible loss of PRC2 has different effects in different cellular contexts. Interestingly, a recent publication reports ANFs have increases in immune signature expression, but are significantly reduced in MPNSTs regardless of PRC2 status.^45^

To focus on potential PRC2-regulated drivers of MPNST, we compared published expression data from 2 PRC2 restored MPNST cell lines to our engineered PRC2 loss iHSCs.^9^ We showed many are likely direct targets of PRC2 in iHSCs, revealing strong candidates for PRC2-derepressed drivers of MPNST transformation. *ARHGAP36* is an oncogene linked to poor prognosis in medulloblastoma and tumor formation in neural progenitor cells.^46^ *CDX2* regulates intestinal epithelial differentiation and is a marker for PRC2-inactivated MPNSTs.^47^ *DTX4* is involved in NOTCH activation.^48^ *TGFA* is a ligand in the EGFR family.^49^ *MRAS* activates the Ras pathway.^50^ *MERTK* is a druggable cancer driver kinase.^51^ We speculate *IL17D* could be a PRC2 derepressed immunosuppressive cytokine.^52^ When comparing genes that are upregulated when PRC2 is restored in Zhang et al., with genes that are downregulated after PRC2 loss, we found only 4 genes overlap. One is *EPHA7*, a receptor tyrosine kinase, a potential tumor suppressor gene.^53^

Some MPNSTs have Notch pathway mutations, including single nucleotide variants in *NOTCH1*.^54^ Whole exome sequencing of patient samples in Suppiah et al. found 3 PRC2-proficient MPNSTs harbor missense mutations in *NOTCH1* and none in ANF samples.^10^ One MPNST sample (M1677) harbors G936S, which maps to exon 18 of NOTCH1 and likely occurs in an EGFR-like domain. Activating mutations in the extracellular EGFR-like domain of NOTCH1 increases receptor sensitivity to ligand binding.^55,56^ Additionally, *Fbxw7*, a negative regulator of NOTCH1 stability, is inactivated by *Sleeping Beauty* transposon mutagenesis in a subset of high-grade mouse SC tumors.^57^

All MPNST lines expressed high levels of all ICDs, generated by NOTCH receptor cleavage.^56^ Previous work indicated higher activation of Notch, assessed transcriptionally, in MPNST PDX models correlates with improved ability to grow in a 3-dimensional microtissue system.^58^ Addition of Notch ICD induced morphological changes in rat SCs, loss of differentiation markers, and transformed cells in a soft agar colony formation assay.^11^ Developmental studies support a link between PRC2 and Notch signaling. PRC2-deficient oligodendrocyte lineage cells failed to differentiate into mature oligodendrocytes and showed upregulation of Notch pathway genes. However, intact PRC2 repressed Notch signaling and allowed proper differentiation to oligodendrocytes.^13^ We speculate this differentiation block phenomenon may also be occurring in SCs. Our studies reveal a new connection between PRC2 and NOTCH that has not been studied extensively in MPNSTs. Notch plays a significant role in the maintenance of cancer stem cell–like cells. Evidence of this has been observed in colorectal cancer with upregulation of *NOTCH1*, *JAG* ligands, and *HES1* resulting in increases in EMT stemness proteins (CD44, SLUG, and SMAD3).^59,60^ There is a need to study how the Notch pathway may drive dedifferentiation of SCs and stemness leading to MPNSTs in NF1 patients.

Though sensitivity is variable, we report nirogacestat, which blocks γ-secretase processing and activation of NOTCH proteins, is capable of inhibiting the growth of MPNST cells and shows strong synergy with MEK inhibition, a common class of drugs investigated in MPNSTs.^61,62^ There is promising work with anti-angiogenesis drugs which block ligand binding to NOTCH receptors (brontictuzumab) and pan-NOTCH inhibitors (crenigacestat).^63^ We predicted NOTCH GOF lines would have higher nirogacestat IC_50_ than luciferase control lines. It is likely nirogacestat has effects beyond Notch signaling as the expression of activated *NOTCH1* or *NOTCH3* only partially protected from nirogacestat and only in ST88-14. For example, γ-secretase also cleaves CD44 and generates a tumor-promoting cytoplasmic cleavage product, commonly expressed in MPNSTs.^64^ Some evidence supports γ-secretase cleaving cadherins which activate WNT signaling.^65^ We, therefore, speculate nirogacestat may be more effective in treating MPNST-G2 which is characterized by WNT hyperactivation.^10^ These ideas must be tested further using in vivo models of MPNSTs. Early studies on the antitumor effect of nirogacestat on desmoid tumors and leukemia cell lines reported increased G_1_ arrest of cells treated with nirogacestat.^66,67^ A slight increase in sub-G_1_ was also noted.^66^ In ST88-14, we observed a sub-G_1_ population after nirogacestat treatment. This may be indicative of apoptotic cells with fragmented DNA. Further, genome instability and polyploidy of high passage cancer cell lines can cause aberrant DNA content staining.^68^ From this, we can speculate nirogacestat might have initially arrested some cells in G_0_/G_1_, but it seems to be primarily inducing apoptosis in MPNSTs which would explain the observed sub-G_1_ population.

In summary, we report upregulation of the Notch pathway after PRC2 inactivation in iHSCs. We show loss of PRC2 derepresses the expression of many genes, including those that activate cancer driver pathways that likely cooperate with RAS-MAPK signaling. These results suggest targeting multiple signaling pathways could be beneficial for MPNST such as MEK plus γ-secretase inhibition.

## Supplementary Material

vdae188_suppl_Supplementary_Materials

## Data Availability

Raw sequencing and methylation probe intensity data can be accessed at https://www.ncbi.nlm.nih.gov/geo/query/acc.cgi?acc=GSE263107 (RNA-seq), https://www.ncbi.nlm.nih.gov/geo/query/acc.cgi?acc=GSE263109 (ChIP-seq), https://www.ncbi.nlm.nih.gov/geo/query/acc.cgi?acc=GSE263127 (Methylome).

## Notes

*Conflict of interest statement*. D.A.L. is the cofounder and coowner of NeoClone Biotechnologies, Inc., Discovery Genomics, Inc. (acquired by Immunsoft, Inc.), B-MoGen Biotechnologies, Inc. (acquired by Bio-Techne corporation), and Luminary Therapeutics, Inc. D.A.L. holds equity in Recombinetics, a genome-editing company, where he also serves on the Board of Directors and acts as a Senior Scientific Advisor. He consults for and has equity in Styx Biotechnologies, Inc. and Genentech, Inc., which is funding some research. The business of all the companies above is unrelated to the contents of this manuscript. Other authors have no conflict of interest to disclose.
