# Supplementary material for: Multiomic analyses reveal new targets of polycomb repressor complex 2 in Schwann lineage cells and malignant peripheral nerve sheath tumors
Source: Neurooncol Adv. 2024 Nov 9;6(1):vdae188. doi: 10.1093/noajnl/vdae188 (PMC11606644; doi:10.1093/noajnl/vdae188)
Supplement: vdae188_suppl_Supplementary_Materials [file vdae188_suppl_Supplementary_Materials.zip › 241029 Supplementary Figures - relabel quant.docx]

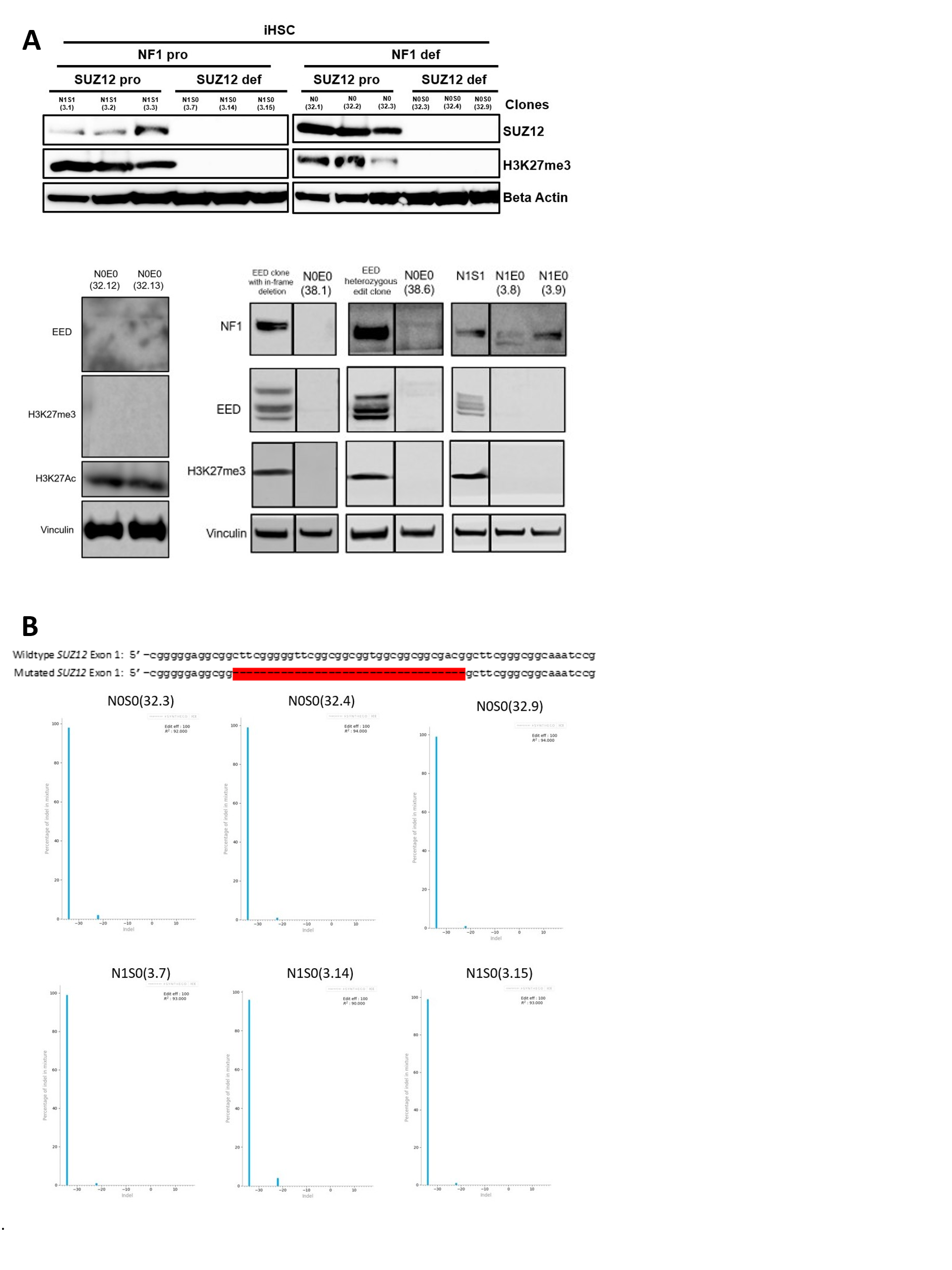

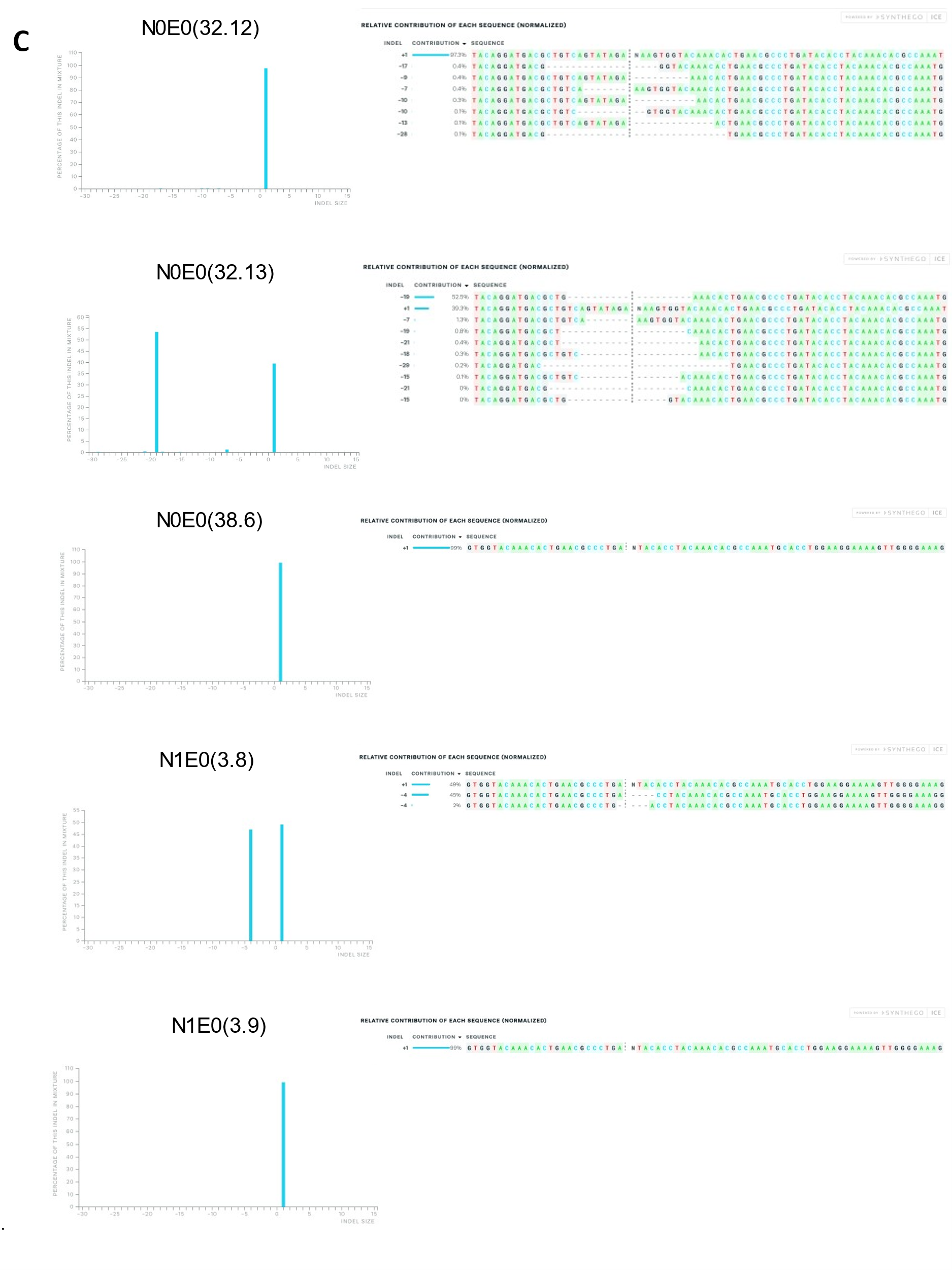


**Supplementary Figure 1: Knockout iHSC generation.** (A) Western blot analysis to confirm successful knockouts. Representative plots for Synthego Inference of CRISPR Edits (ICE) analysis of (B) SUZ12 edited lines and (C) EED edited lines.


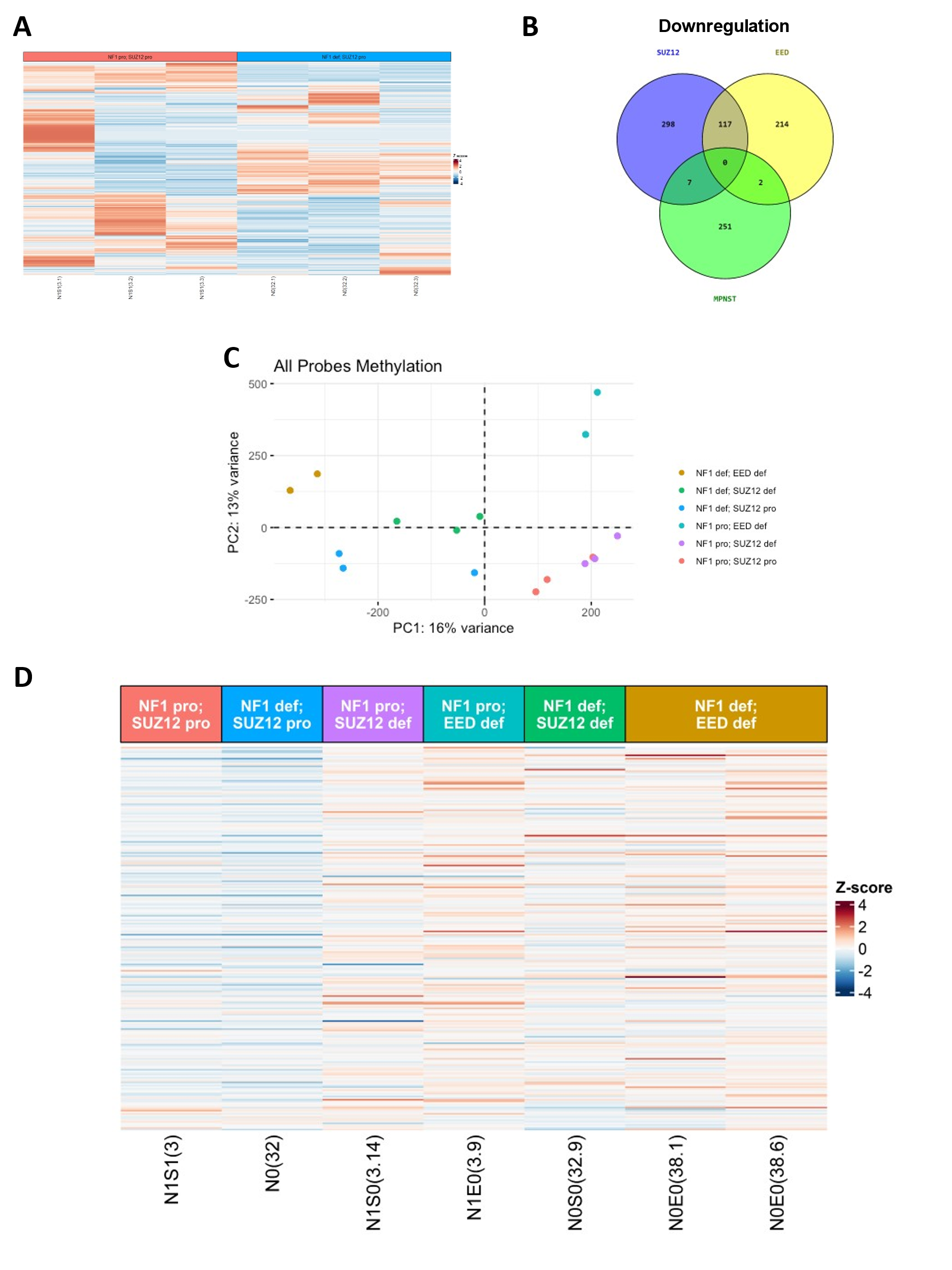


**Supplementary Figure 2: Differential expression analysis and methylome clustering of engineered *NF1* and *SUZ12*-deficient iHSCs.** (A) Loss of *NF1* leads to 1439 differentially expressed genes in human SCs. (B) Overlap of downregulated differentially expressed genes from MPNSTs, NF1 and PRC2 deficient iHSCs lead to 0 overlapping genes, but 117 genes are downregulated when PRC2 is lost in iHSCs. (C) Engineered iHSCs cluster by methylome status. (D) Heatmap of 354 differentially abundant proteins that overlap with 1,327 differentially expressed genes.


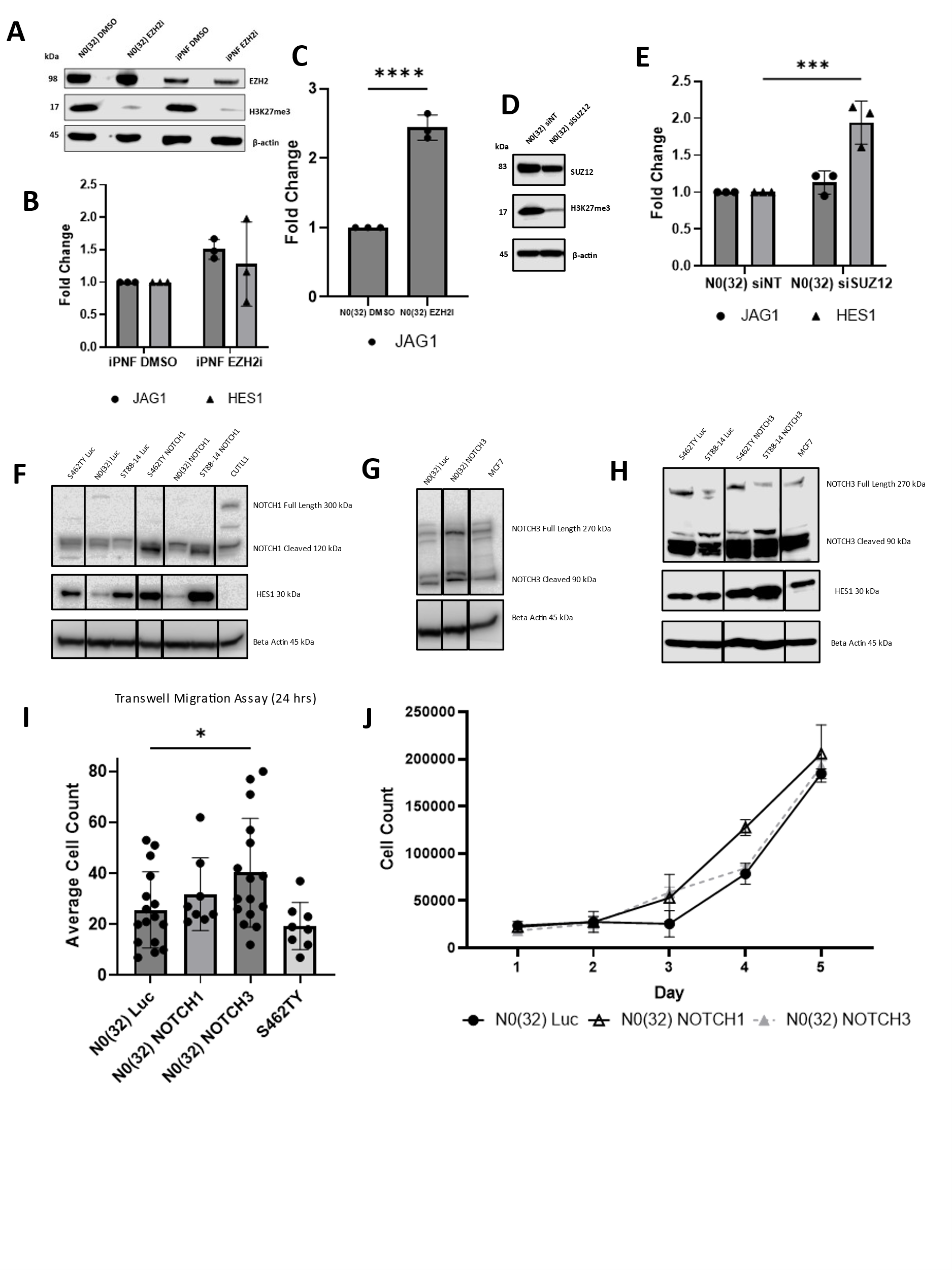


**Supplementary Figure 3: PRC2 knockdown leads to increased JAG1 and HES1 expression and NOTCH3 overexpression increased migration in N0(32) cells.** (A, D) Western blotting shows lines treated with EZH2i and siSUZ12 leads to loss of H3K27me3. (B-C, E) RT-qPCR of upstream and downstream markers of NOTCH signaling on generated transient cell line models (pooled siRNA or EZH2 inhibitor treatment). Western blots show GOF of (F) NOTCH1 and (G, H) NOTCH3 were successful in S462TY, ST88-14, and N0(32). (I) Transwell migration shows a significant increase in migration in N0(32) NOTCH3 GOF lines. (J) Proliferation of N0(32) GOF lines in a 6-well plate over 5 days. 20,000 cells were plated on Day 0. 2-way ANOVA of Luc vs NOTCH1 yields a p value of 0.0146 (*) and Luc vs NOTCH3 yields a p value of 0.39 (ns).


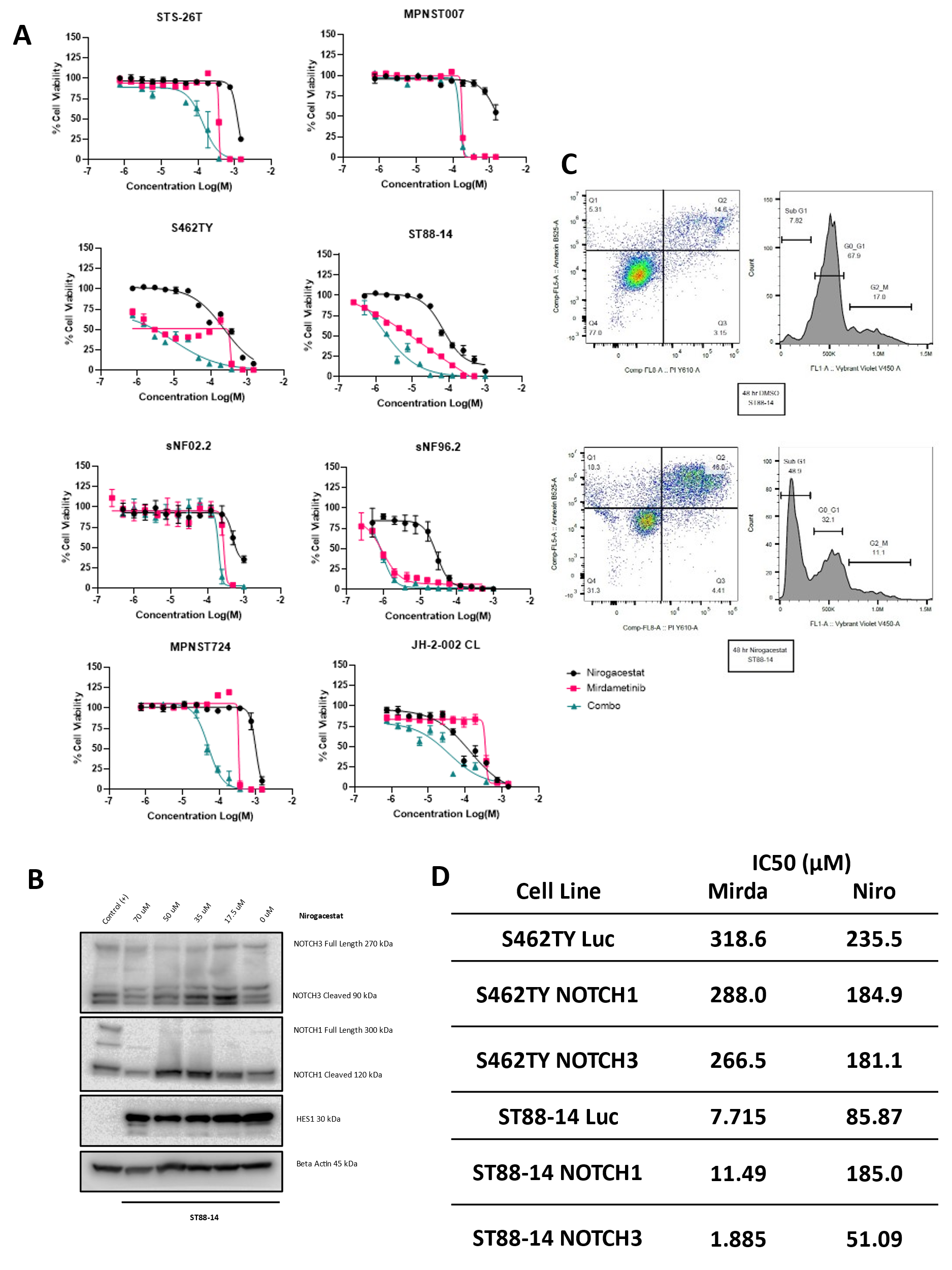
 **Supplementary Figure 4: Nirogacestat and mirdametinib in combination may be an effective treatment for MPNSTs.** (A) Dose-response curves for the cell lines summarized in Table 1. (B) Small molecule inhibitor studies found the IC50 of nirogacestat in ST88-14 to be around 70 uM (Table 1) which was confirmed via Western blotting NOTCH1 and NOTCH3. Positive control for NOTCH1 is CUTLL1 and MCF7 for NOTCH3. (C) Flow cytometry of nirogacestat treated ST88-14 lines showed increased apoptosis and a sub-G_1_ cell population. (D) Table of IC50 values of each GOF line. Experiments independently repeated at least twice.


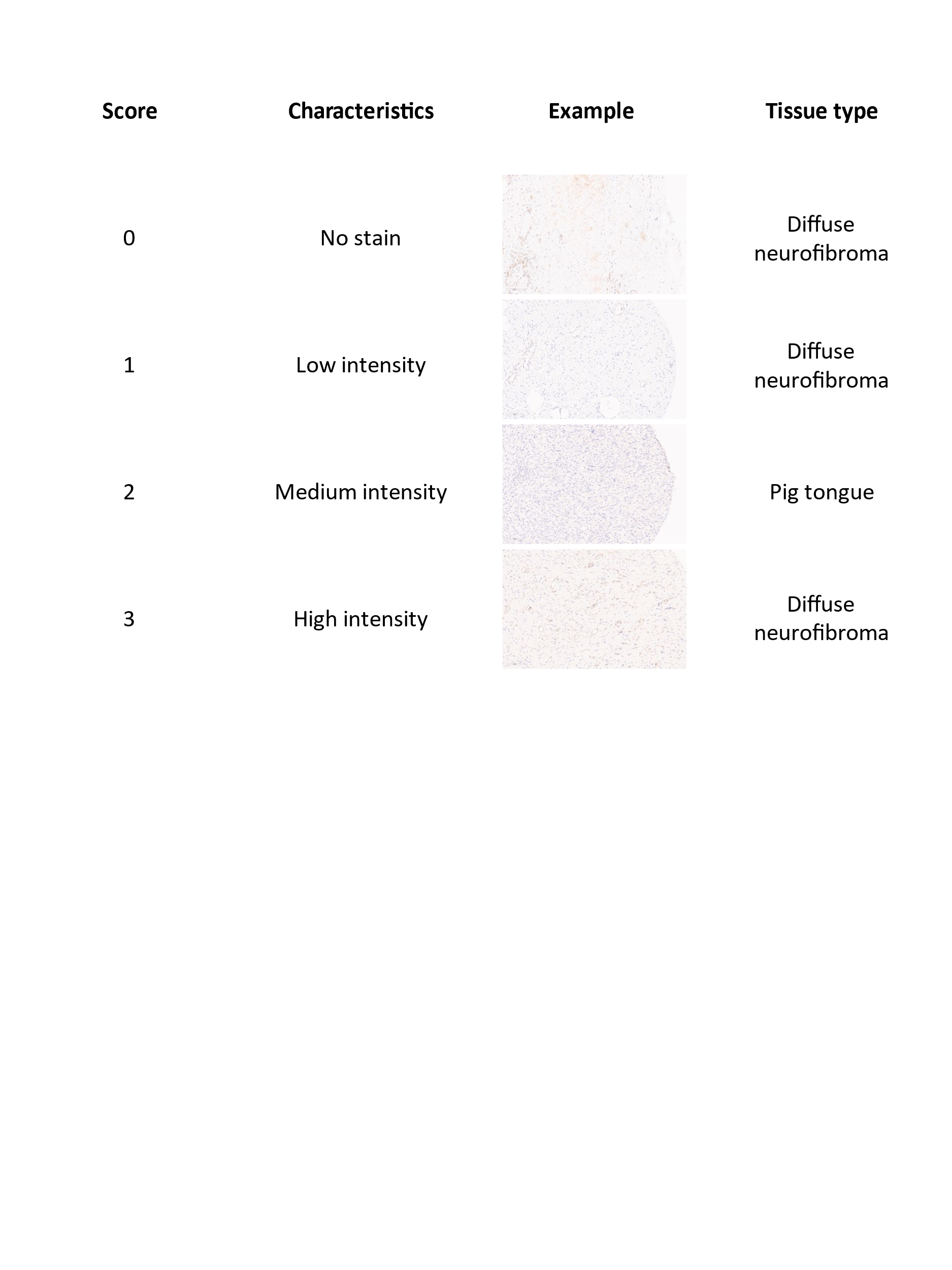


**Supplementary Figure 5: Examples of TMA staining intensities**.


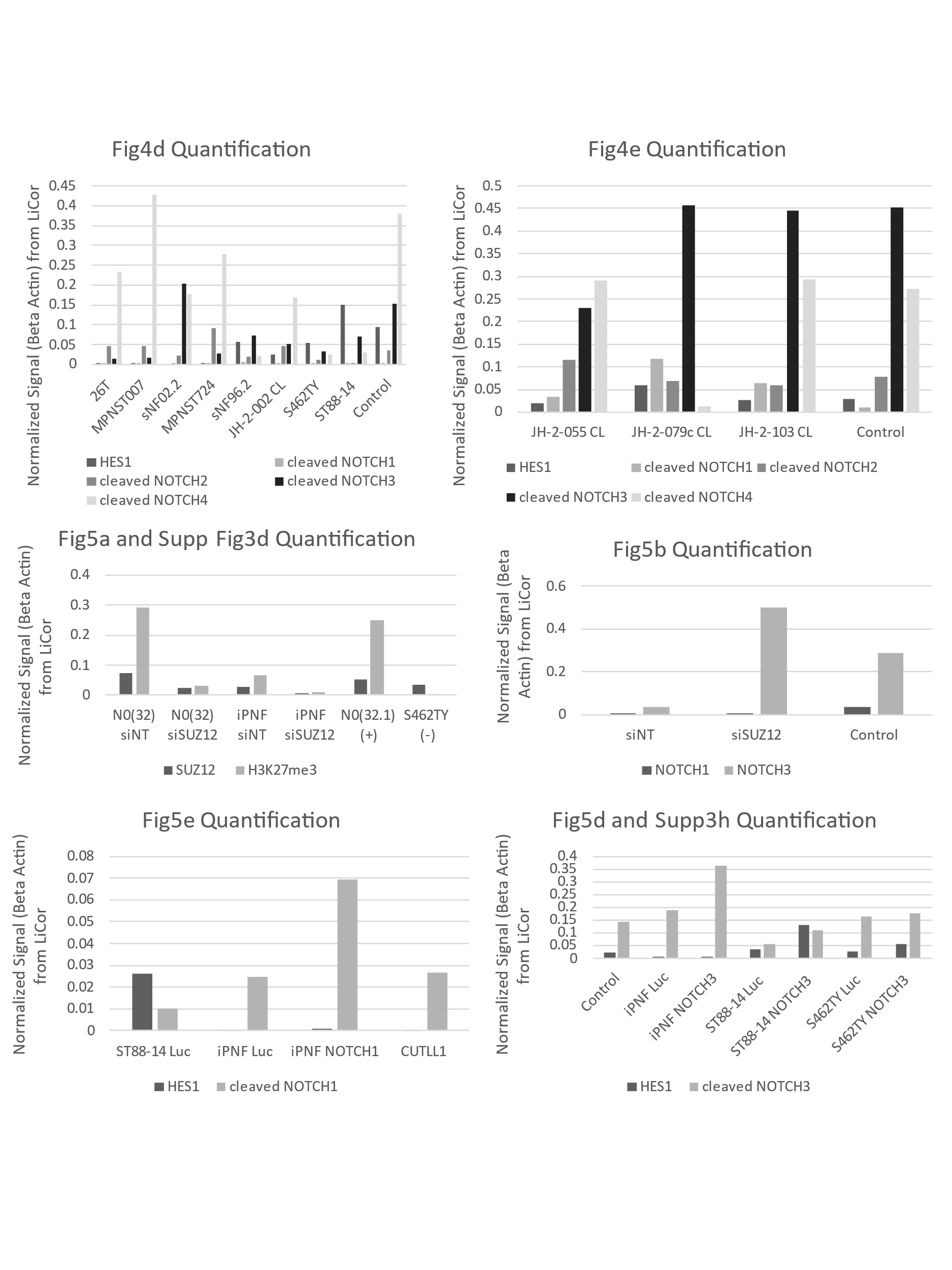

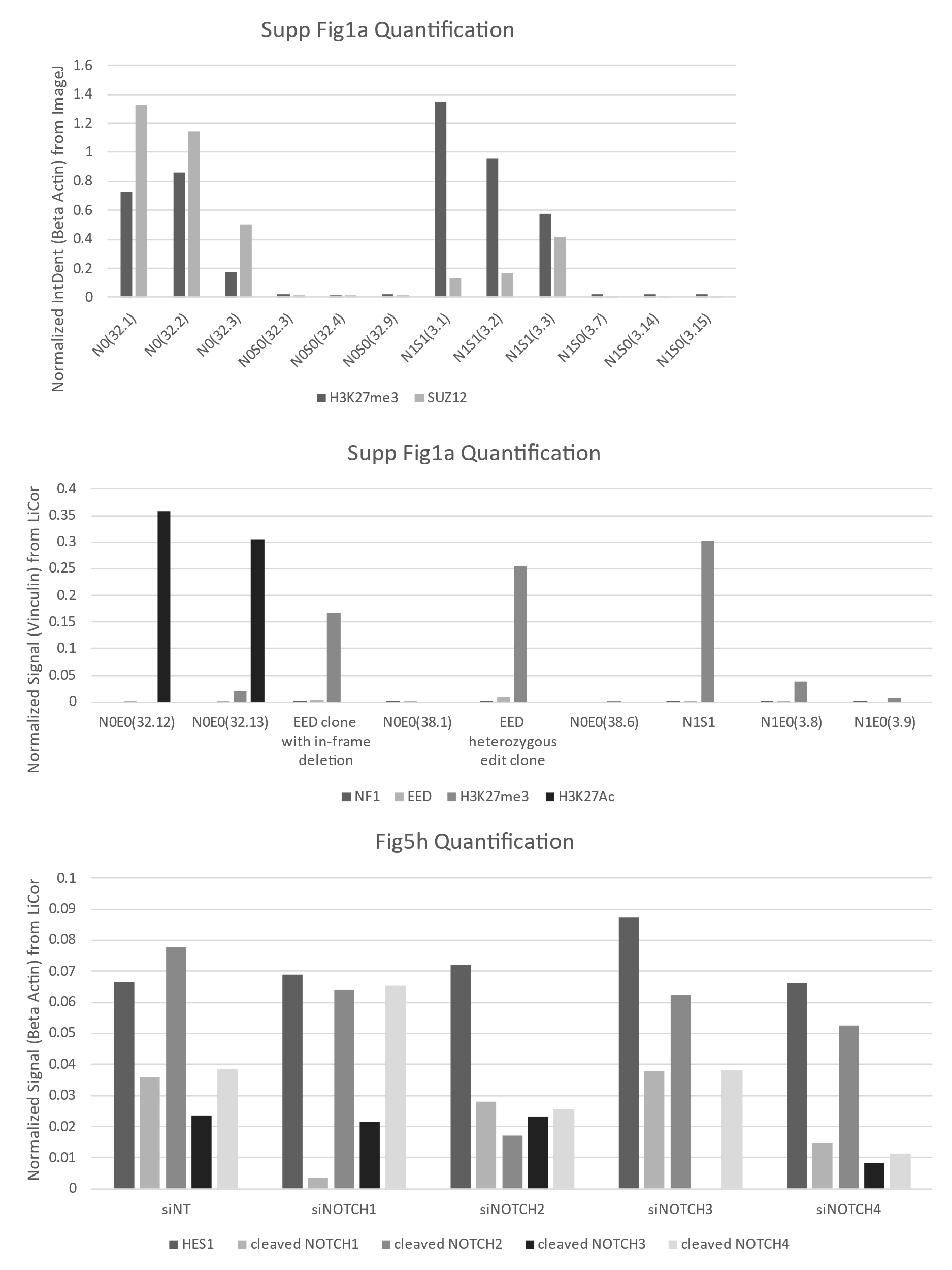


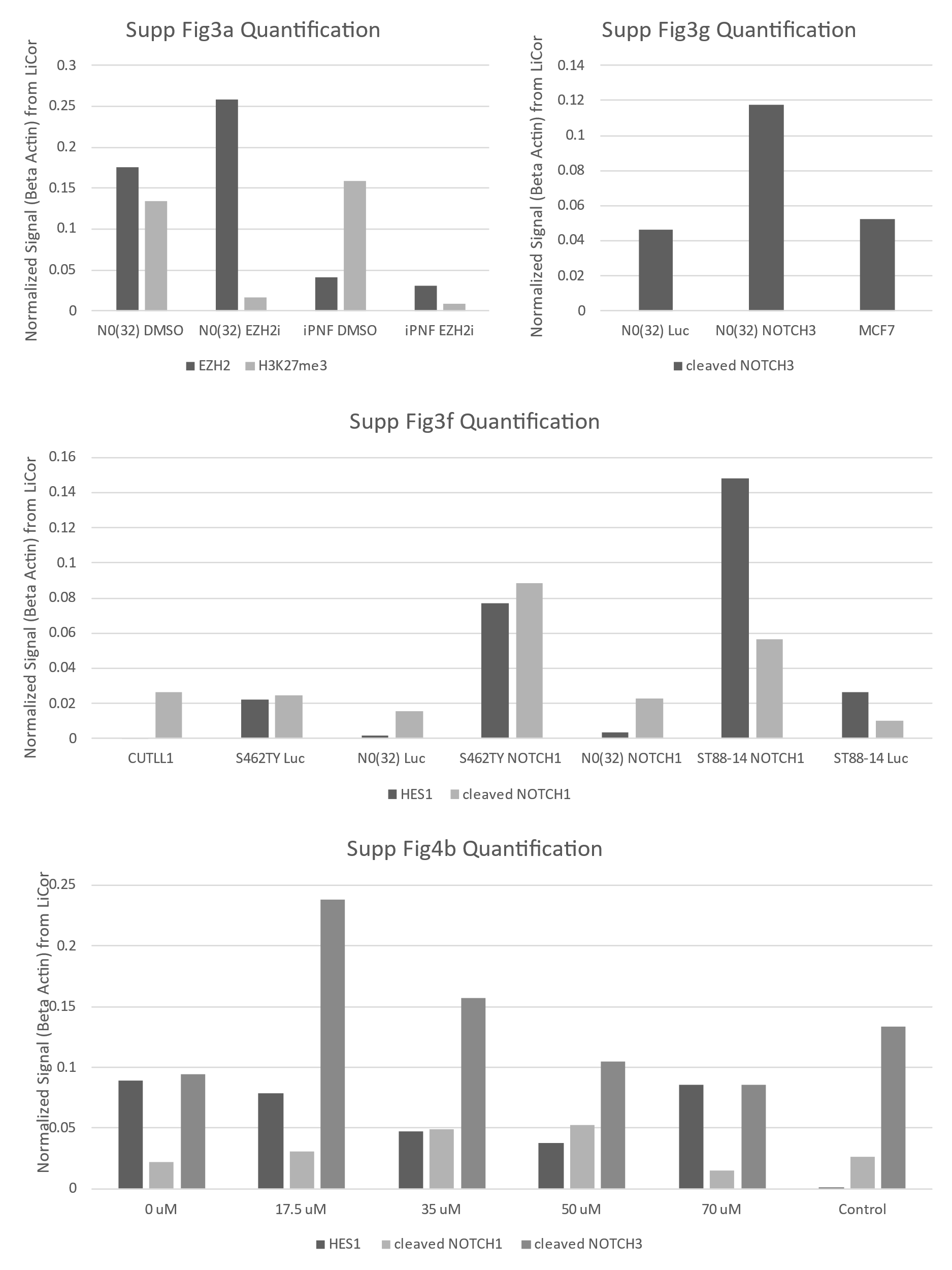


**Supplementary Figure 6: Western blot quantifications.**
